# Buffalo Medical and Surgical Association

**Published:** 1889-08

**Authors:** 


### *BUFFALO MEDICAL AND SURGICAL ASSOCIATION.*

REGULAR meeting of June 4, 1889,—the President, Dr. ALVIN A. HUBBELL, in the chair.

The following question was discussed :

*In view of the various causes of insanity, do the insane receive the best medical and surgical treatment under the present system of asylum management? If not, how can such treatment best be secured?*

DR. CHARLES A. RING—All that relates to insanity as one of the questions of social science confronting the public, to the insane as a class, and to the individual patient, insane, arouses the most intense interest, anxiously searches for instruction and guidance, and demands attention for immediate relief.

Accompanying, side by side, are suspicion and distrust—the one a malign shadow to the community, holding many under its influence; the other destroying that confidence which all hospitals and asylums should rightfully possess.

As indicated, the community is three-fold: the layman, with or without the ties relating to the patient; the family physician, watching over the interests of the patient at home, and the various officials and boards protecting, as far as able, the patient in his commitment; and third, those having the care or the custody of the patient. What are the relations of each class, their responsibilities, and their rights? The right of the community to knowledge of its local institutions is absolute, and is formally recognized in the reports of officials. These reports are generally regarded as complete, as far as they go, but technical. Practically, this knowledge is obtained through the press, which generally correctly states the conditions of affairs; and the opinions formed thereon accurately represent the community. It is a sad comment that is raised by the fact that of all the surreptitious introductions of reporters into asylums, not one has produced reports which are creditable to the institutions. Recently, Nellie Bly, for the New York *World;* Charles W. Beck, for the Chicago *Times;* and George W. Symonds, for the Philadelphia *Inquirer*, have given the public information which will be used with profit.

From knowledge comes the direct display of power possessed; and wisdom determines the responsibility of institutions.

Second. The family physician and the official boards.

Reliable statistics show that, of the insane committed to and admitted by State asylums of this State, over fifty per cent. are either chronic or incurable cases. Here is certainly a most deplorable exhibit, a large part of which is placed directly upon the family physician, with what fairness I shall leave them to determine. In many chronic cases, and in those of acute character where delay has existed, the public is obliged to exercise the right of protection.

This right of protection, which any citizen possesses, the public has not formally extended to the insane individual, so that he may himself be protected from the ravages of disease. To state the case mildly, I will call this the advanced opinion of many, which I will not bring into conflict with the home treatment of the patient.

It is estimated that it costs ten times as much to maintain a chronic case through its average of twelve years as it does to cure an acute curable one.

If the public accepts this advanced opinion, it must furnish hospitals for the insane as unexceptionable in public opinion as are the general hospitals of any large city.

The relations of the physician to the public are many and complex. Those which look toward the enlightening, prevention, and protection are especially interesting. Meetings like the present, with its subject-matter for presentation and discussion, should be considered as especially valuable, and the information obtained be made available.

Public demand has led to the creation of various offices and boards having relations with the dependent classes. These officers have accomplished much good work, secured many reforms, and advocated advanced opinions. The most prominent are the State Board of Charities, and the State Charities Aid Association, with their local delegations. The recent change from one to three State Commissioners in Lunacy, with the many requirements of the Act, shows the constant advance.

Third. Institutions for the insane.

These should be of two distinct classes: one of hospitals for the acute curable cases or of doubtful character; the other, custodial.

The fact that only about twenty-five per cent. of cases admitted recover, should lead to distinct and prominent individualization of the patient who is susceptible of improvement to a marked degree, or of entire recovery.

There should be a very full resident medical staff, and also consulting physicians and surgeons. There should be numerous attendants, who should be well paid, well instructed and disciplined, and who should be, socially, the peers of the patient under care.

The custodial cases require surroundings that shall be homelike and yet afford every facility for rendering themselves as nearly self-supporting as possible.

It has been stated by good authority that the institutions for the insane of this country are not equal to those of similar classes in Europe. It is well that the defects are kindly pointed out and criticisms made. The suggestions should be received in the same spirit, and promptly carried into execution.

Where doubt and distrust exist, those mingling with the public should inform those responsible for the institution, so that its course may be changed and it enter upon the career of usefulness intended. If, after the utmost endeavor, the highest results are not attained, steps should be taken to change the system.

Without reference to the causes of insanity, I am of the opinion that the insane—curable, incurable, and chronic—do not receive the best treatment and care. As indicated, I believe that public confidence must be restored. There should be mutual respect and coöperation.

The present system of management must be modified in many particulars to secure the desired results.

Dr. James W. Putnam—To answer the first question affirmatively, is to assert that a human system is perfect, not susceptible of further improvement. Any one who has watched the progress of political science has noticed how one system has in time given place to others. In medicine we have abundant opportunity of seeing how ephemeral a history many medical fashions have had.

Methods of treatment formerly universally practised are now practically abandoned, either because the type of disease had changed or because new and better methods had superseded the old.

This has been the case in all branches of medicine. Surgery has been revolutionized by the introduction of antisepsis. Gynecology has had its fashions. Ophthalmology, since my graduation, has been greatly modified by the introduction of cocaine.

When we come to study the history of institutions for the care of the insane, we find as marked changes in this as in all other departments of medical thought. Ever since Pinel's broad humanity removed the chains from the insane, modification in the methods of treatment has been the rule. And who among us shall say that now we have reached the top, and now we have the best medical and surgical treatment under the present system of management? To say that, is to arrest all attempts to improve. It is mental stagnation. Let us consider the situation for a moment.

A large institution for insane men and women, numbering in our asylum about 460 persons—to take care of them is a superintendent, whose duties are executive and medical, and to aid him in this work are three assistants.

A patient on entering the institution is carefully questioned as to his hereditary and clinical history and personal habits, religion, etc. Pulse, temperature and respiration are noted, and the patient is sent to the proper ward. If the past history of the patient is important, how much more so is the present condition? It would be of great value if, in addition to the record already taken, we should be able to read the report of an examination of the urine, the lungs, the heart, the eyes, and the report of a gynecological examination of all female patients with symptoms of pelvic disease.

Such an examination would be of immense value, as it would bring to light any local conditions which affected the general health, and so give better opportunity to build up the general health in a more definite manner than by giving the constitutional bitter tonics, and iron and cod-liver oil.

As the State now provides, it is impossible to give patients the thorough special examination outlined. The assistants, skilled in diagnosticating the mental state, are, with very few exceptions, able to make accurate examinations of the eye. More of them are able to make gynecological examinations.

Nevertheless, it is unsafe to assume that in all asylums we will always find physicians holding the appointments, who have the proper training for such special work as has here been outlined.

The benefit to the insane resulting from the gynecologists' treatment has been briefly told in a valuable paper by Prof. Reed, read before the Alumni Association of Niagara University, April 9, 1889. Although I agree with Prof. Reed in his opinion, I do not go so far as to adopt his substitute. He says: "This brings me to my final suggestion: Abolish the superintendent and assistant system, and instead appoint a staff made up of representative specialists, supplemented by an adequate corps of house physicians."

This, to my mind, would not be a step in advance, but, on the contrary, one in retrograde. There is a great power of control, great wealth of experience, great moral tone, given to an asylum by a superintendent like Dr. J. B. Andrews, of the Buffalo State Asylum for the Insane. I believe in appointing medical superintendents, but I believe in relieving them of their executive duties.

To conduct the general treatment of the patients in the asylum, I believe the appointment under civil service rules of young men to the positions of assistants inspires confidence in the public and insures good service. Knowing from personal experience what the requirements of an asylum are, I know that the house staff of a general hospital is entirely inadequate to the demands of an asylum.

My proposition would be to appoint a surgeon, an ophthalmologist, and a gynecologist, who should visit the asylum once a week and examine all new cases, and let them institute such treatment as they found necessary.

These appointees should be paid by the State, their work should be special, and would in no way interfere with the general treatment by the asylum resident staff. In this way we would insure against such charges as that made by Dr. Reed: "The staff wait for these cases to die to find out what is the matter with them," and we would secure to the patients all the benefits of specialism.

Dr. H. R. Hopkins—It is not my purpose, in any remarks which I shall offer to the Association this evening, to attempt to traverse the entire subject embraced in the question under discussion; but simply to emphasize certain impressions which have gathered in my mind during twenty years of study and observation upon this matter, impressions which have crystallized into convictions and beliefs by recent review of the literature of this subject.

One of the gentlemen who preceded me has very properly called attention to the fact that, in the nature of the case, perfection in the treatment of insanity is not attainable. It is not, therefore, necessary for me to detain the Society in any elaboration of this apparently axiomatic truth. As we, therefore, can probably easily agree that improvement in the treatment of insanity may be achieved, it is to our particular purpose to give a moment's thought to the lines upon which, or over which, this improvement may come.

The relations of the unfortunate insane to the people of the State of New York, and to the profession of this State, first took shape by an Act of the Legislature passed in the year 1788, entitled "An Act for Apprehending and Punishing Disorderly Persons." It is worth while in passing to stop for a moment to remark that this law of 1788 was founded upon the English law enacted in the year 1744. The condition of the public mind at this time is illustrated by a simple reference to this act of 1788. The "disorderly persons" which were to be apprehended and punished included fortune tellers, tramps, mountebanks, common prostitutes, and the insane.

The conditions of the insane, under this unfortunate procedure, were such as at this day can scarcely be conceived. The law empowered two Justices of the Peace to apprehend the insane, to confine them in suitable places,—if necessary, in chains,—in order that the public might be protected from them. The thought that the insane person was a sick person requiring treatment does not seem to have had any position in the public mind of this date. In the year 1836, the Medical Society of the State of New York, representing the medical profession of the time, memorialized the Legislature in terms at once accurate, emphatic and eloquent, calling attention to the fact that an unfortunate class of our citizens, the insane, demanded in the name of humanity recognition and suitable treatment.

As a result of this memorial, laws were enacted establishing State asylums and hospitals for the insane, and from this time, to the medical profession at least, the insane man has simply been a sick man requiring intelligent treatment addressed to his malady. Previous to this time, as the law plainly indicated, the insane man was a strange compound, partly criminal, partly demoniacal, and altogether too horrible for anything save chains and the dungeon.

This is the year 1889, and yet it is necessary to call the attention of this Society, and of the medical profession which this Society represents, to the fact that in the management of the insane, we have retained certain demoniacal residua. Our statutes and our usages still provide, that before an insane man can be subjected to suitable treatment, the machinery of the courts has to be invoked, and he has to be committed, to an asylum to be sure, and yet this asylum is abundantly supplied with locks, bolts, bars, handcuffs, straight jackets, camisoles, and various methods of treatment hardly compatible with the idea that the insane is only a patient needing medical treatment. Is it not time, Mr. President and gentlemen, that the medical profession asserted itself and recognized, in fact as well as in theory, that the insane is not demoniac, but only ill? Could anything be more absurd than for a medical man of this day to apply to the courts for permission to treat a case of inflammation of the eye, to place a patient suffering from inflammation of the eyes in that condition where injurious influences are to be excluded from him; or, in case of a fractured femur, to invoke the machinery of the law to enable him to place such restraint upon the patient as experience has demonstrated is necessary to his proper recovery? For myself I can see no lack of similarity between the requirements of one suffering from an inflammation of the eye, or suffering from a fractured femur, and one suffering from mental disease; and it seems to me that it is for the medical profession to assert that it has no conception of an insane man other than as a patient requiring relief, and that the medical profession is in a situation to demand that there shall be no circumlocution, no embarrassments placed between the most enlightened treatment and the suffering citizen in need of such treatment.

This brings me to a suggestion which I would like to make to the Society, and that is, that it is not expedient that the care and management of the insane of any community be placed in the hands of a few individuals of the medical profession. In my judgment the members of the medical profession at large are the safest custodians for this responsibility, and I would suggest for the consideration of this Society the propriety of recommending that every hospital have attached to it a suitably prepared ward for the reception of the acutely insane. To these wards, so equipped, patients should be admitted with no more circumlocution than pertains to the admission of patients to the surgical ward, the lying-in ward, or any other department of a well-equipped hospital. If statistics have ever established any fact in medicine, they have established the truth that early treatment in the history of insanity exercises the most hopeful influence over the case. One of the speakers who preceded me has very appropriately called attention to the fact that of the patients received at any of our asylums a large percentage are chronic cases. In these cases the best opportunity for cure has been lost, and what is the reason why so many cases only reach the asylum when the acute stage has passed? I will not undertake to state to this Society all the reasons which operate in these cases. I will simply refer to one, and that is the stigma which rests upon the judicial commitment of an individual to an insane asylum. This stigma is well known and dreaded, and it is from this stigma—this asylum stain—that the acutely insane should be relieved. I am absolutely convinced that if the insane could be sent to properly appointed hospitals for the same reason that the sick in general are sent to such hospitals, the proportion of insane coming under early treatment would be manifestly increased, and that most expensive and hopeless class of dependents, the chronic insane, would be correspondingly diminished.

In regard to the question of the improvement of our present method of treatment of the insane, I would also suggest that, in every State institution, the diseases of the insane, the varieties of insanity, should be taught to the medical students and to the medical profession who wish to avail themselves of such opportunities. To my mind it is another instance of demoniacal residuum that the wards of our asylums are closed to the extent they now are. Let us look upon these wards as places for the treatment of the sick, and they immediately become suitable fields for clinical observation and instruction. In my judgment, the importance of this step in the effort to secure an improved treatment of the insane cannot be over-estimated. The staff of any insane asylum which shall also be the faculty to teach the diseases of the insane, will not be found wanting in the methods of diagnosis, or in the application of remedies for the relief of their patients. Again, no visiting committee, no committee of inspection will more thoroughly introduce the outside world to the life of the asylum than will the clinic of medical students gathered from all quarters of our population. To my mind, the importance of this step is manifest alike to the managers of our asylums and the unfortunates in whose interests our asylums are established and maintained.

One more thought to which I would like to invite the attention of this Association, in criticism upon the present treatment of the insane, is that the line of promotion in the staff of the insane asylum runs away from contact with the patient. I can illustrate what I mean in this direction, perhaps, no better than by referring to the conditions surrounding the young practitioner of medicine. During the first year of this young gentleman's experience, he does well if he sees patients to bring him in $500 income. With good behavior on his part, this income steadily increases year by year, little by little, until, after ten, fifteen, or twenty years, he comes to enjoy a full practice. During this period of waiting, ample time has been given him to study, to reflect, to observe, to assimilate, and to become competent. In the asylums, matters are very differently arranged. The young man, fresh from the lecture-room, takes a position of junior assistant at the asylum, and is placed in almost absolute control of 200, 300, or 400 insane. To be sure, he has over him a senior assistant and the superintendent; but, in my judgment, the fault of the system lies in that the members of the staff nearer the head have more and more of their time engaged in purely administrative work. In many instances, in the case of the superintendent, his entire time is so engaged. Not only do the affairs of the institution for which he is responsible demand of him a large portion of his time in administrative work, but his opinion is frequently sought as an expert in important cases before the courts; and in other ways his engagements are so numerous that the individuals under his care, who are expected to receive, and who should receive the immediate benefits of his experience and ability, see but very little of him. In my judgment, the time will never come when the practice of medicine can be done after the manner of wholesale business; when the superintendent of an asylum can say: "Let us treat our cases of acute mania after this order, of acute melancholia after that plan, of chronic melancholia after another fashion; let us surround our dements with such-and-such a treatment and atmosphere;" but, in my judgment, it will remain the fact that effective work can only be done by the study of individual cases, where the strength and the efficiency of the individual practitioner is brought to bear upon the individual peculiarities of a given case. It would seem to me that the medical profession is in a situation to demand, in the presence of facts, that the purely executive and administrative work of our asylums be done by individuals other than the medical staff.

As a result of some little experience with various asylums throughout the country, and from the impression gathered by some little study of the subject, your speaker is persuaded that there is too much idleness, too few employments, too little incentive to labor, exercised in the management of the chronic insane. In the first year of my professional experience, spent in one of the leading insane asylums in the country, the impression was firmly fixed in my mind that a suitable incentive would lead to large returns in the way of labor on the part of the insane, by seeing the squad which daily left the asylum to labor upon the farm and in the fields of the place, under the incentive of a daily ration of tobacco. The question of cottage farms, of diversified and various labors,—labors which in their nature are light and can be made attractive,—are questions upon which the medical profession should have clear ideas.

A further criticism, which my observation suggests, upon the treatment of the insane is that, in the management of our asylums in general, too much use is made of such remedies as chloral and bromide of potassium, the various narcotics, and sedative remedies, to the exclusion of more rational means of medication; such, for instance, as are included in an intelligent hydro-therapeutics. It would seem that this latter system of medication is one singularly adapted to great institutions, where the facilities are readily at hand. If it were the custom of our various asylums to state in their annual reports the amounts of the various narcotic and cerebral sedatives used in the different institutions per year, I think it would be but a short time before some enterprising institution would make the experiment of the habitual use of the hot bath, the cold bath, the hot and cold douche, the hot-air bath, the steam bath, and the various appliances of modern hydro-therapeutics, to the end that great economy was observed in the use of the more powerful drugs, and at least an equally good showing in the column of recoveries and improvements.

In conclusion, I would call attention to the fact that no system of treatment of the insane can ever be thoroughly successful which does not pay great heed to the character of the attendants who coöperate with the medical staff. In this connection, it gives me pleasure to cite the instance of the training-school for nurses instituted in our State asylum. Such a step should receive at the hands of the medical profession every possible commendation and support. In this connection, I would also refer to the desire on the part of the women of our community to take a more active interest in the management of our asylums for the insane. In my judgment, it is a move in the right direction, and one that the medical profession should further at every opportunity. So long as the insane require nursing, will women continue to furnish the most desirable material for nurses. To them only can we look for that degree of patience, of tact, of devotion, that aptitude for consecration which this work demands. I should be proud to see this Society recommend to the public that the work of nursing the insane be placed in the hands of women whose lives are devoted and consecrated to this work. It would be suitable for society to encourage such women by every possible commendation and support, and it would seem to me that the efficiency and the perpetuity of the service would be enhanced by demanding of candidates for this work vows of celibacy and vows of obedience.

Dr. A. W. Hurd, referring to a point of a previous speaker, spoke, first of the limited applicability of "home treatment" of the insane. In cases of wealth and proper surroundings only can violent patients be taken care of at home. Friends must make of their house a hospital for the insane for the time being, with trained nurses and constant medical attendance, if they are to do as well for the patient as the best-equipped hospitals of to-day. While lunacy retains its dangerous features,—dangerous to the individual and to society,—so long will institutions be a necessity for their care and restraint.

Further work in the present line of improvement and betterment of hospitals for the insane seems to be the method in which we are to expect the best results, rather than in new and startling innovations.

A resident medical head, a man of responsibility, experience, and training, is a necessity for the proper discipline and working of an asylum; and no staff of consulting physicians, visiting at intervals, can have that intimate knowledge of patients and nurses which is necessary to secure the most efficient service and results. In this, as in other matters, the man who devotes his life to one study and object is better fitted than he whose time is largely taken up with other interests. But the commendable practice is rapidly growing, especially in asylums located in cities, of appointing a consulting staff of specialists. In the Buffalo asylum the city specialists are frequently and freely called on, when their services are needed. In this respect, institutions located near cities have great advantage over county asylums, or those remote from medical centers, who have not ophthalmologists, gynecologists, surgeons, etc., within easy call. Those county asylums in which the number of patients is not sufficient to warrant the employment of a resident medical officer, should be abolished, and their patients put under constant medical supervision. Another step in the right direction is the greater knowledge demanded of candidates for appointment as assistant physicians. The following questions of a recent Civil Service examination in this State for such positions indicates that the successful candidate must be possessed of an average if not *more* than ordinary ability :

1. In what part or parts of the brain must a lesion be situated in order to cause motor aphasia?

2. In what part to cause sensory aphasia?

3. In what part to cause left lateral homonymous hemianopsia?

4. What is meant by the term "Jacksonian epilepsy?"

5. In what part of the brain must a lesion be situated which causes attacks of convulsive movements commencing in the right foot and extending up and involving the right leg and thigh, and then extending to the right arm and right side of the face, unassociated with loss of consciousness, but associated with a steadily increasing weakness and awkwardness of the right leg?

6. How would you determine the point of the skull to trephine in order to reach the lesion producing the symptoms described in the preceding question?

7. What are the symptoms which lead you to consider a patient insane?

8. What are the early symptoms of "General Paresis of the Insane?"

9. What are the lesions of "General Paresis of the Insane?"

10. Mention some drugs or poisons which produce transitory attacks of insanity, and some of the characteristic symptoms of each variety.

11. What relation do attacks of epileptic insanity bear to the convulsive attacks, and what symptom is common to both?

12. What points of difference are there between the mental depression occurring in melancholia and that which may occur in chronic delusional insanity?

13. Give the differential diagnosis between hematocele, hydrocele, varicocele, and scrotal hernia, and the treatment of each.

14. What is "Potts' fracture?"

15. How would you reduce a dislocation of the shoulder?

16. On what theory is the use of antiseptics in surgery based?

17. Give the differential diagnosis between small-pox, scarlet fever, measles, and chicken-pox.

18. Give the physical signs of the three stages of pneumonia.

19. Give exact directions for the disinfection of typhoid fever stools.

20. Give the treatment for acute articular rheumatism.

21. How would you treat a case of placenta prævia.

22. Give the pathology and treatment of puerperal eclampsia.

23. Give the physiological effects of belladonna; of digitalis; of chloral.

24. What is the poisonous dose of each of the drugs mentioned in the last question?

25. Give the various tests for sugar in the urine, and explain the chemistry of each.

Politics should not, as in some States, enter into asylum management, and no staff of physicians, devoting themselves to this specialty, should feel that their labors were to terminate in case the dominant political party should fail at the next election. Such a State policy does not tend to attract the best medical talent to the specialty.

The care of the insane is improved by the instruction given in the training-schools for attendants which are now being established so generally in hospitals for the insane. The placing of female nurses on the convalescent male wards is an advantage, and worked for the welfare of the patients. The speaker believed this system was only fitted, however, for the quiet and *not* for the disturbed wards, and could not at all agree with the view previously advanced, that no male nurses be employed in asylums.

As bearing on the subject of the welfare of the insane, the speaker spoke of the legislation on this subject during the past Winter, and read extracts from the new Lunacy Commission Bill, which has become a law, and the new bill for the commitment of the insane, which had passed both houses and awaited the Governor's signature.[1] In the new Commission Bill, that clause requiring every physician in the State,

---

1. Note.—The Commitment Bill has failed to become a law.

qualified by a judge as an examiner in cases of lunacy, to file such qualification with the Commissioners at Albany, was read as being of general interest to the profession.

The advantages of certain features of the new Commitment Bill, should it become a law, were commented on, viz. : the greater judicial character of the commitment, each patient being committed on the order of a court, and the clause which provides for the voluntary commitment of those numerous cases of nervous and mild mental disease who feel that they need the care and treatment of an institution, but shrink from the process of being examined and committed as insane, as is now necessary.

The subject was further discussed by Drs. Stockton, Crego, Hill, O'Brien, and Long.

The committee appointed to present a memorial on the death of Dr. Edward Tobie, reported as follows:

"In full vigor of health apparently, and in the midst of a large practice, Dr. E. Tobie died on the 12th of May last, so suddenly that the painful event could scarcely be realized by his many friends.

"Dr. Tobie was known as an indefatigable worker, with a devotion to his patients rarely excelled. He was a good citizen, true and generous, and as a physician well-informed, skilful, and successful, commanding as such the most implicit confidence of his patrons, to whom his demise is a great loss sincerely lamented.

"To his many friends, both lay and professional, the Buffalo Medical and Surgical Association desire to express their high estimation of his value as a physician, and to testify their sorrow in his sudden and untimely death. To his family they tender their unfeigned and heartfelt sympathy.

"Signed,

"JOHN HAUENSTEIN,
"JOHN D. HILL,
"F. A. BURGHARDT."

